# Adenoid cystic carcinoma of the Bartholin’s gland is easily misdiagnosed: A case report and literature review

**DOI:** 10.1097/MD.0000000000031744

**Published:** 2022-11-11

**Authors:** Wenhui Wang, Hao Chen, Hualei Guo, Lei Chen, Miaoping Zhu, Yingjia Zhu

**Affiliations:** a Department of Pathology, Hangzhou Women’s Hospital, Hangzhou, Zhejiang, China; b Department of Ultrasound, Hangzhou Women’s Hospital, Hangzhou, Zhejiang, China; c Department of Radiology, Hangzhou Women’s Hospital, Hangzhou, Zhejiang, China; d Department of Gynecology, Hangzhou Women’s Hospital, Hangzhou, Zhejiang, China.

**Keywords:** adenoid cystic carcinoma, Bartholin’s gland, misdiagnosis, MYB, MYB-NFIB, pathology

## Abstract

Adenoid cystic carcinoma (ACC) often occurs in the major and minor salivary glands and other sites containing secretory glands, while ACC of the Bartholin’s gland (BG-ACC) in the vulva is rare and easily misdiagnosed.

**Patient concerns:**

A 58-year-old female was referred to our hospital for further valuation of a mass occurring on the left side of her vulva. In the other hospital, the beginning of the period, local ultrasound showed a vulva mass, which was suspected to be a Bartholin’s gland cyst. Mixed neoplasms were considered in some biopsies. When transferred to our hospital, virtuous tumors were considered by ultrasound and magnetic resonance imaging. Pathology initially considered benign hyperplastic active tumor or borderline tumor.

**Diagnoses:**

Histological, immunochemical, and molecular tests confirmed a diagnosis of BG-ACC, negative surgical margin, without lymphatic metastasis.

**Interventions:**

Extended excision of the mass at left labia majora plus left inguinal lymph node dissection was performed.

**Outcomes:**

The patient received surgery therapy, no recurrence was observed during a 18-month follow-up period.

**Lessons:**

Due to its lack of specific characteristics in clinical, ultrasound and imaging, it is easy to be misdiagnosed, Due to its rarity and nonspecific clinical, radiologic and ultrasonographic manifestations, BG-ACC can be easily misdiagnosed. And its pathomorphological features overlap with other benign and malignant tumors occurring at vulva, BG-ACC can be easily misdiagnosed, and diagnosis by puncture biopsy is extremely difficult. Use of paraffin sections to identify tumor growth characteristics, combined with immunohistochemical findings, is the key to the diagnosis of ACC. In rare sites, MYB gene split are helpful in making a definite diagnosis.

## 1. Introduction

Adenoid cystic carcinoma (ACC) is mostly found in the salivary glands but can also occur in the esophagius, breast, lungs/bronchus, prostate, and lacrimal glands. Primary ACC of the Bartholin’s gland (BG-ACC) is rare, accounting for about 10% of adenocarcinoma of Bartholin’s gland and 0.1% to 5% of malignant tumors of the vulva.^[1]^ Most patients with vulvar ACC are aged between 50 and 67 years (average 59 years).^[2]^ BG-ACC usually presents as painful, itchy, or swollen vulva, although it can be asymptomatic in some patients. Therefore, there is a possibility of misdiagnosis and delayed treatment.^[3]^ Clinically, BG-ACC has a high risk of local recurrence and distant hematogenous metastasis. Prompt diagnosis of ACCs at rare sites is particularly important for the treatment and prognosis. Due to limited literature, genetic findings within BG-ACCs are rarely reported. Herein, we present a representative case from our center and perform a literature review for BG-ACC.

## 2. Case presentation

**Figure 1.**
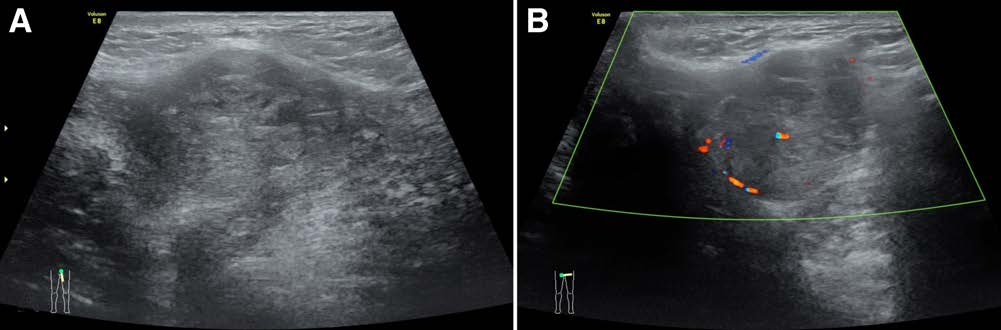
(A, B) Ultrasonography showing in homogenous echoes under the labia majora.

**Figure 2.**
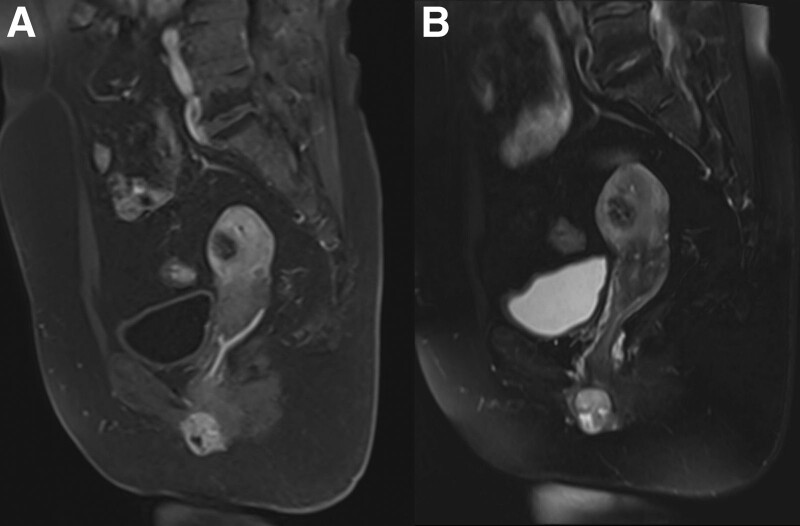
(A, B) Magnetic resonance imaging (MRI) showing that there was a circular signal anomaly on the left side of the vagina, with a clear boundary and high signal hybridity on T2W1.

A 58-year-old woman had a quail egg-sized, painless mass accidentally palpatedon her left vulva. In outer court, local ultrasound of the superficial tissue revealed a vulvar mass, with the presence of fluid sonolucent areas. The lesion was suspected as a Bartholin gland cyst, and an incision and drainage procedure was performed, during which the mass felt firm. A partial biopsy was performed for pathology examination. Pathology considered a tumor of cutaneous adnexal origin, and its morphological and immunohistochemical findings were consistent with those of a mixed tumor. The consultation diagnosis at another hospital was “eccrine sweat gland tumor, with partially active hyperplasia.” She then visited our hospital. On physical examination, there was a subcutaneous firm nodule sized 3.0 cm × 2.0 cm × 2.0 cm in the left labia majora, with a distance of 3cm from the midline. The lesion had clear border, poor mobility. Ultrasound showed a heterogeneous echogenic focus of about 3.2 cm × 2.2 cm × 2.1 cm in the subcutaneous muscle layer of the left labia majora, with its upper edge adjacent to the lower external edge of the left pubic bone (Fig. 1). Magnetic resonance imaging demonstrated a round-like mass with a circular abnormal signal on the left wall of the lower vaginal opening. The lesion had a clear boundary, showing equal T1 signal, with small flakes of high signals inside (Fig. 2). B ultrasonography and influence showed no malignant pointer. Intraoperative exploration showed that the tumor was firm and solid, located in the fascial layer. It had clear border and poor mobility. Extended excision of the mass at left labia majora plus left inguinal lymph node dissection was then performed.

**Figure 3.**
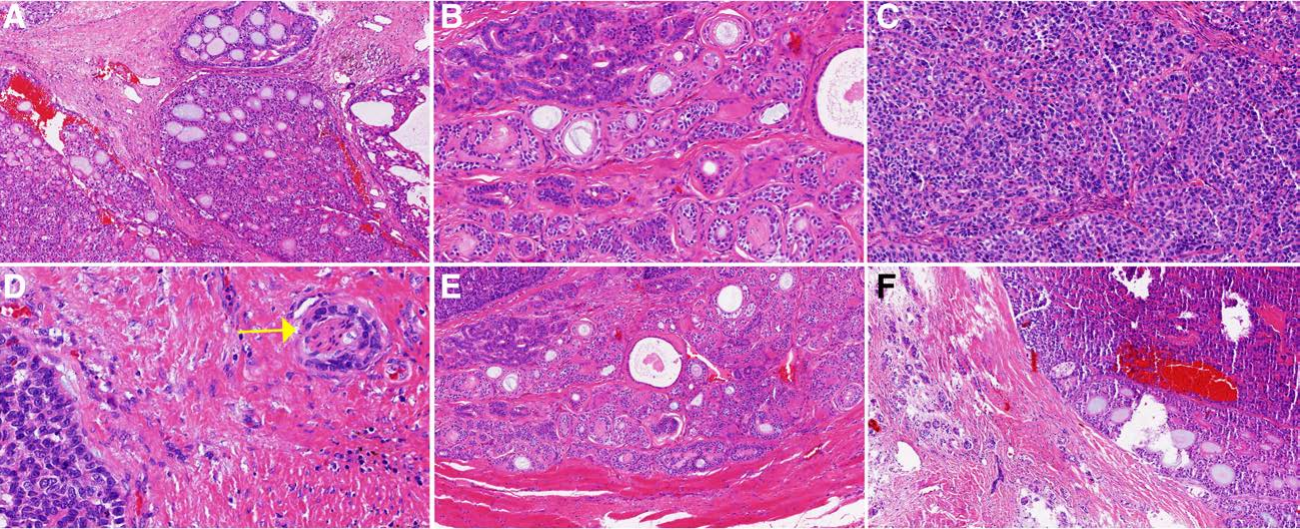
Histology of Bartholin’s gland adenoid cycstic carcinomas (Hematoxylin and eosin stain); (A) Cribriform pattern with lumens containing basophilic mucin (original magnification × 100); (B) Tubulai pattern (original magnification × 200); (C) Solid growth pattern (original magnification × 200); (D) Tumor cell nets are seen surrounding a nerve fiber (marked by arrows), indicating perineural infiltration (original magnifications × 200); (E) Tumor cells infiltrate skeletal muscle (original magnification × 200); (F) Normal gland and ducts were observed around the tumor tissue (original magnification × 100).

**Figure 4.**
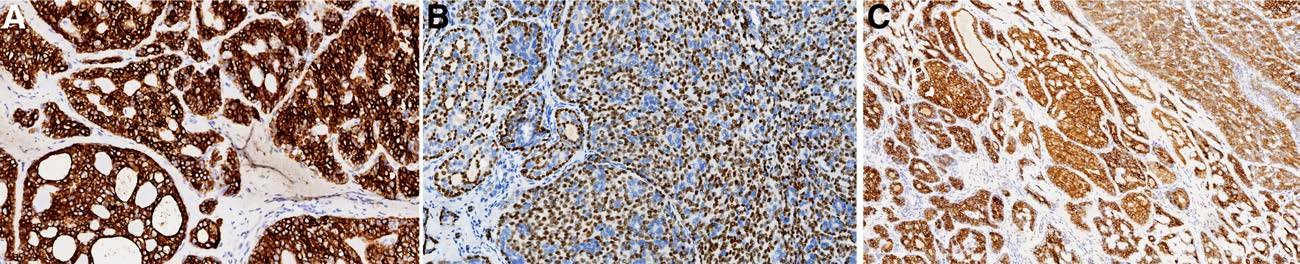
Immunohistochemical results (original magnification × 200). (A) Glandular epithelium is positive for CK7; (B) Myoepithelium/base cells are positive for P63; (C) Diffuse positive for CD117.

**Figure 5.**
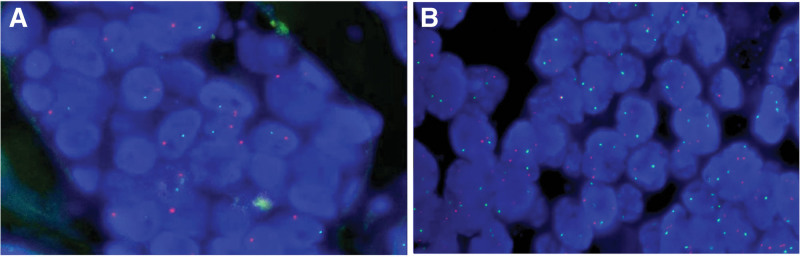
(A) FISH for MYB with a break-apart probe in tumor cells displays a rearrangement involving MYB (separation of green and orange sign); (B) No change was observed in MYB-NFIB gene copy number by FISH assay. FISH = fluorescence in situ hybridization.

Gross pathology revealed that the mass was 3.3 cm × 2.6 cm × 2.5 cm in size, with grayish-yellowish cut surface and fine texture. Bleeding was seen in some areas. The lesion was seemingly enveloped on its surface, and its borders with the surrounding tissues were clear. Microscopically, the tumor was nodular, with fibrous tissue as its pseudo-envelope. Local infiltrative growth of the tumor was observed outside the pseudo-envelope. The growth patterns were mainly cribriform and tubular, whereas solid lamellar pattern was seen in focal area. The tumor cells were consistently of medium size, consisting of basal-like, glan-dular, and myoepithelial cells, although double-layered cell structure was also visible. Secreted mucus or red-stained homogeneous cylindrical material was seen in these glandular cavities or cribriform holes of different sizes (Fig. 3 A–C). They had round or ovoid nuclei, with rare pathologic mitosis and sparse cytoplasm. There was nerve invasion in the humble (Fig. 3D). In a small number of paraffin blocks, tumor invasion into skeletal muscle was visible in the focal area when the blocks were cut deeply and consecutively (Fig. 3E). A small amount of residual Bartholin’s glands were seen in the focal area around the tumor (Fig. 3F). The immunohistochemical findings included (Fig. 4): CK7+/CK14+/EMA + glandular epithelial cells; p63+/S-100+/SMA + myoepithelial/basal cells; diffusely positive for CD117 and Sox-10; wild-type P53+; and Ki-67 index about 15%. Fluorescence in situ hybridization (FISH) analyses showed rearrangements of the MYB locus (separation of green and orange signals) (Fig. 5A). FISH molecular studies using break-apart probes showed no deletion or amplification of MYB-NFIB fusion gene in tumor cells (Fig. 5B). The final pathological diagnosis was “BG-ACC, negative surgical margin, without lymphatic metastasis.” During the 18-month-follow-up, no local recurrence or distant metastasis was found.

## 3. Discussion

BG-ACC is a rare slow-growing tumor. It originates from the Bartholin’s gland in most cases but may also come from the apocrine glands in a small number of cases, with its surface covered with intact skin. More than 150 BG-ACC cases have been reported worldwide. Because the initial clinical manifestations of BG-ACC in most patients are similar to those of benign Bartholin’s gland cysts or abscesses and the imaging findings are often nonspecific, their diagnostic performance is often limited, there are challenges such as misdiagnosis, delayed treatment, and disease progression affecting the quality of life. Jones et al reported a case of ACC that was initially diagnosed as a Bartholin’s cyst, leading to further misdiagnosis and mistreatment for 2 years.^[4]^ Yoon et al reported a case of a clinically diagnosed Bartholin’s abscess that was surgically resected after 8 months of ineffective treatment for abscess, with a final pathological diagnosis of ACC.^[2]^ Ultrasound and magnetic resonance imaging are helpful only when the tumors are poorly circumscribed and infiltrating the surrounding tissues.

In addition, BG-ACC is rarely seen in routine pathologic work-ups, and the unique structures of the vulva and the special growth pattern of the disease make BG-ACC easy to be confused with other benign and malignant tumors, especially during pathological diagnosis of biopsy and frozen sections. Fine-needle aspiration cytology has been found to be useful for ACC at other sites, although false negatives have been reported.^[5,6]^ Hughes et al investigated 6249 salivary gland tumors in 2005, with a high false negative rate of fine needle aspiration cytology of 33% for ACC.^[7]^ The possibility of misdiagnosis or missed diagnosis can be high if the biopsied tissue sample is featured predominantly by solid and tubular structures and has neither typical cribriform structures nor definite nerve or peripheral tissue infiltration (as in the present case). For patients aged 40 years or older with clinically suspected Bartholin’s cyst or abscess, a puncture biopsy is needed at least to rule out the possibility of malignancy if the mass is found to be firm and poorly mobile during physical examination, drainage, or surgery.

BG-ACC has the same histologic pattern as ACCs occurring in the parotid gland and other sites. Ueda et al performed a retrospective pathological review of cases diagnosed as salivary gland ACCs treated by surgical resection in 15 hospitals in Japan, and found that the diagnoses were corrected in 21 cases (6 corrected as benign tumors and 15 as other malignancies).^[8]^ ACC is rare in most pathology departments, especially in obstetrics and gynecology centers. The pathologic diagnosis of ACC in some specific sites remains extremely challenging. Copeland et al developed the criteria for the pathologic diagnosis of primary vulvar Bartholin’s gland tumors: the tumor is located in the anatomic area of the Bartholin’s gland, which is located deep in the labia majora, and the skin on the surface of the tumor is generally intact; migration transition from normal Bartholin’s gland to neoplastic area is observed under microscope; residual glandular tissue can be seen in the surrounding tissues of the tumor; in situ carcinoma components are present in the glands and ducts of Bartholin’s gland, which is consistent with the origin of the tumor in Bartholin’s gland; and no recent concomitant tumors of similar histological type at other sites.^[9]^ Histologically, the tumor has a typical cribriform glandular-like cystic pattern, often surrounding an acellular gap that is filled with PAS-positive substances or granular basophilic substances. Most glandular lumens are not true glandular lumens but extracellular spaces containing overlapping substrate substances and tumor-produced mucin. Small true lacunae are also formed in the tumor, and in some tumors there are also solid lamellar and glandular duct-like structures formed by morphologically similar cells. Microscopic diagnosis of ACC requires the presence of both pseudocystic and true glandular cavities.^[10]^ The tumor is featured by the infiltration into the perineal body, and one of its distinctive biological features is nerve invasion; therefore, many patients were present with pruritus and burning sensation before the appearance of a palpable mass. However, this symptom was not evident in our case. If the tumor and its surrounding tissues are adequately sampled but no nerve infiltration is found, the diagnosis of ACC needs to be made with caution, especially when the tumor is located in uncommon sites.

The ultrastructure of ACC is characterized by pseudo glandular cavities, intercellular spaces, large amounts of substrate-like substances, and true glandular cavities.^[11]^ Tumor cells include intercalated duct cells, myoepithelial cells, secretory cells, and multipotential reserve cells.^[12]^ There is almost no difference in histogenesis among ACC and benign mixed tumors, and the differential diagnosis is mainly based on the classical cribriform structure as well as the invasive growth in nerves and surrounding tissues. Immunohistochemical staining of tumor cells in ductal structures expressed the same staining characteristics as intercalated ductal cells [positive for keratin, CEA, S-100, and CD117 (c-Kit)], and staining of cells surrounding the pseudocystic lumen suggested myoepithelial differentiation (positive for S-100 and actin and heterogeneously positive for keratin).^[13–15]^

The etiology and mechanisms of ACC remain unknown, especially for ACC occurring in the lower genital tract. It has been reported that 7 of 14 patients with vulvar ACC were pregnant at the time of diagnosis, suggesting that pregnancy is one of the risk factors and hormones may play an important role in its pathogenesis.^[9,16]^ Although ACC-containing mixed cervical cancers are associated with high-risk human papillomavirus, high-risk human papillomavirus infection is not associated with simple vulvar or cervical ACC.^[17]^Cytogenetic analysis of vulvar ACC revealed complex chromosomal changes in chromosomes 1, 4, 6, 11, 14, and 22, with structural and unbalanced rearrangements resulting in loss of chromosomal fragments 1p31-qter, 4q22-q28, 6p12-qter, 11p11.2-pter, 14q24-qter, and 22q13-qter.^[18]^ MYB-NFIB fusion transcript was detected in 14(44%) of 32 paraffin-embedded ACC specimens; in 2 of 3 patients with vulvar ACC, the recurrent t (6;9) (q22–23; p23–24) translocation was associated with the MYB-NFIB fusion protein.^[19]^ In a recent study, 6 of 9 vulvar ACC cases demonstrated NFIB separated signals. Of these 6 cases, only 2 cases were positive for a MYB rearrangement, which was also confirmed by a positive MYB-NFIB fusion gene. The NFIB-associated gene rearrangement is a common genetic event in vulvar ACC. A novel MYBL1- NFIB gene fusion involving t (8;9) translocation have been reported in 35% of salivary gland-derived t (6;9)-negative ACCs.^[20,21]^ Tumors with the t (8;9) translocation are associated with high MYBL1 expression, which may confer novel oncogenicity in ACC. In a whole-genome sequencing study, 5 different rearrangements were detected in 28% of salivary gland ACCs, involving NFIB but with an intact MYB. Novel NFIB fusion partners identified in that study included MYBL1, MAP3K5, RPS6KA2, MYO6, and RIMS1.^[22]^ It was also found that fusion transcript-specific RT-PCR for MYB-NFIB and MYBL1-NFIB and ordinary split FISH assays for MYB and MYBL1 were less sensitive.^[23]^ In addition, KRAS and KDM6A mutations, were detected respectively in 2 patients with BG-ACC, in whom 160 cancer-related genes were analyzed using targeted genetic sequencing. Which contributes to new targeted gene therapies, including the use of BET and HDAC inhibitors.^[24]^ Further studies on the specific roles of these chromosomal translocations may provide new insights into the oncogenic driver and potential therapeutic target of vulvar ACC, thus promoting the personalized, precise treatment of this disease.

Surgery remains the primary choice of treatment for BG-ACC. In a review by Alsan et al, simple and radical vulvectomy accounted for 54% and 46%, respectively. Recurrence was seen in 35% of patients with positive margins and10% of those with negative margins.^[25]^ It was reported that local excision resulted in a recurrence rate of 69.8% and a positive margin rate of 48%, while the extensive vulvectomy caused a recurrence rate of 42.9% and a positive margin rate of 30%.The prognostic and therapeutic values of lymph node dissection remains unknown.^[26,27]^

It has been widely recognized that the prognosis of BG-ACC patients is closely related to the surgical margins, and a negative margin is a key indicator for clinical cure. However, local recurrence and distant metastasis have been reported even in patients with negative surgical margins, with the lungs as the most commonly involved organ,^[25,28,29]^ and metastases to bone, kidney, liver, and other organs have also been reported in a small number of patients.^[30,31]^ Due to the rarity and the long-term survival of BG-ACC, a large cohort study or a randomized study on this disease is not feasible, thus it is difficult to assess accurately the efficacy of a specific therapy.

## 4. Conclusions

BG-ACC is a rare disease, and misdiagnosis and delayed diagnosis may occur in both clinical and pathologic diagnoses. Although BG-ACC grows slowly, it is characterized by nerve invasion, surrounding tissue invasion, distant metastasis, and high rates of positive surgical margins that are invisible to the naked eye. Vigilance is required when clinical symptoms are vague and pathologic diagnosis is confusing. Radical resection with adequate resection range is essential. Conventional treatments include extensive resection and adjuvant radiotherapy (if needed). Patients with high risk factors may undergo adjuvant radiotherapy. Local recurrence and long-term distant metastasis may occur, and thus longterm follow-up is required. With a better understanding of the tumorigenic mechanisms of BG-ACC, molecular testing and gene-targeted therapy are expected to provide more precise treatment of this rare tumor.

## Author contributions

All authors contributed to the preparation of the manuscript. Wenhui Wang contributed to the concept, design and drafting of the manuscript. Hao Chen was involved in editing the final draft of the manuscript. Hualei Guo contributed to obtaining the microscopic images. Lei Chen was involved in ultrasound diagnosis, and provide ultrasound pictures and report. Miaoping Zhu was involved in radiological imaging and provided the radiological images and report. Yingjia Zhu was involved in clinical surgical management of the patient and contributed with a critical revision of the manuscript. All authors revised and approved the final manuscript.

**Conceptualization:** Wenhui Wang.

**Data curation:** Wenhui Wang, Hualei Guo.

**Formal analysis:** Wenhui Wang, Hao Chen, Yingjia Zhu.

**Investigation:** Wenhui Wang.

**Methodology:** Wenhui Wang, Hao Chen.

**Resources:** Lei Chen, Miaoping Zhu.

**Writing – original draft:** Wenhui Wang.

**Writing – review & editing:** Yingjia Zhu.
